# Occupation, smoking, and chronic obstructive respiratory disorders: a cross sectional study in an industrial area of Catalonia, Spain

**DOI:** 10.1186/1476-069X-5-2

**Published:** 2006-02-14

**Authors:** Ángeles Jaén, Jan Paul Zock, Manolis Kogevinas, Antonio Ferrer, Albert Marín

**Affiliations:** 1 Centre d'Estudis Epidemiològics sobre la SIDA de Catalunya (CEESCAT), Crta de Canyet s/n, 08916 Badalona, Spain; 2 Respiratory and Environmental Health Research Unit, Institut Municipal d'Investigació Mèdica (IMIM), Dr. Aiguader 80, 08003 Barcelona, Spain; 3 Hospital de Sabadell. Consorci Hospitalari Parc Taulí, Parc Taulí s/n 08208 Sabadell, Spain

## Abstract

**Background:**

Few studies have investigated the independent effects of occupational exposures and smoking on chronic bronchitis and airflow obstruction. We assessed the association between lifetime occupational exposures and airflow obstruction in a cross-sectional survey in an urban-industrial area of Catalonia, Spain.

**Methods:**

We interviewed 576 subjects of both sexes aged 20–70 years (response rate 80%) randomly selected from census rolls, using the ATS questionnaire. Forced spirometry was performed by 497 subjects according to ATS normative.

**Results:**

Lifetime occupational exposure to dust, gases or fumes was reported by 52% of the subjects (63% in men, 41% in women). Textile industry was the most frequently reported job in relation to these exposures (39%). Chronic cough, expectoration and wheeze were more prevalent in exposed subjects with odds ratios ranging from 1.7 to 2.0 being highest among never-smokers (2.1 to 4.3). Lung function differences between exposed and unexposed subjects were dependent on duration of exposure, but not on smoking habits. Subjects exposed more than 15 years to dusts, gases or fumes had lower lung function values (FEV_1 _-80 ml, 95% confidence interval (CI) -186 to 26; MMEF -163 ml, CI -397 to 71; FEV_1_/FVC ratio -1.7%, CI -3.3 to -0.2) than non-exposed.

**Conclusion:**

Chronic bronchitis symptoms and airflow obstruction are associated with occupational exposures in a population with a high employment in the textile industry. Lung function impairment was related to the duration of occupational exposure, being independent of the effect of smoking.

## Introduction

Chronic Obstructive Pulmonary Disease (COPD) is one of the most important respiratory causes of mortality and morbidity in developed countries. COPD is characterized by the presence of persistent airflow obstruction that is typically progressive and include different clinical conditions as chronic bronchitis and emphysema [1]. Smoking is the main preventable risk factor for the development of this disease [2]. Occupational exposure has been related to several respiratory diseases, mainly with bronchial asthma and COPD [3-6]. Although several epidemiological studies have found an association between occupational exposure to dust, fumes and gases, and chronic bronchitis and airflow obstruction, the role of occupational exposure in the development of chronic airflow obstruction is still controversial. One of the main reasons for this is the difficulty to assess the independent effect of occupational exposure and smoking [7,8]. Both risk factors are determined by amount as well as duration of exposure. Even after adjusting associations between occupational exposure and COPD for smoking status, a residual confounding effect could remain. In addition, an interaction between occupational exposures and smoking has been described [9]. For all these reasons it has been argued that a separate analysis of smokers and non-smokers is preferable [9].

On the other hand, when evaluating the effect of occupational exposure on lung function it is important to take into account the duration of lifetime exposure. Cumulative exposure to dust and increasing working years in specific jobs have been associated with a steeper decline in FEV1 [10,11]. Although workforce-based studies allow an accurate measure of occupational exposures, selection bias is more likely to occur due to the healthy worker effect [12]. We assessed the association between lifetime occupational exposure and airflow limitation in a cross-sectional survey of an urban-industrial area of Catalonia, Spain with a high prevalence of occupational exposures.

## Methods

### Population and questionnaire

Methods of this study have been described in detail elsewhere [13]. Briefly, a random sample of 722 men and women aged 20 to 70 years was selected from a general population census-roll, among residents of the catchment's area of Parc Taulí Hospital (Sabadell, Barberà, Badía, Santa Perpétua, Castellar and Sant Llorenç). The sample was stratified by municipality and was proportional to the number of inhabitants in each municipality. The sampled urban-industrial area in Catalonia, northeastern Spain included 161,585 inhabitants between 20 and 70 years of age. Eighty subjects could not be localised or had deceased, and of the remaining 642 eligible individuals 66 refused to participate. Thus, the final study population consisted of 576 subjects (response rate 80%). Between June 1993 and December 1994, 501 subjects were interviewed face-to-face and 75 by telephone. We used the standardized questionnaire of respiratory symptoms of the American Thoracic Society (ATS) [14], translated into Catalan and Spanish. Subjects that had smoked 20 or more packs of cigarettes during their lifetime, or at least one cigarette per day during one year, one cigar/week during at least one year, or eight pipe tobacco bags were regarded smokers. Subjects that had been smokers in the past according to the above definition, but quit smoking at least six months before the survey, were regarded ex-smokers.

### Spirometric measurements

We used a portable computerized spirometer (Datospir-92, Sibelmed, Spain) calibrated daily with a syringe of 3 litres and monthly with an explosive decompressor. All spirometric measurements were performed exclusively by one of the authors (AJ), according to the ATS normative. Subjects that did not provide at least two technically acceptable maneuvers after eight attempts were excluded. Bronchodilator test was performed 15 minutes after spirometry by administration of 0.2 mg albutamol metered-dose inhaler (MDI). Values of the forced expiratory volume in 1 second (FEV_1_) were compared with sex-, age- and height-specific reference values determined for Mediterranean populations [15].

### Definitions of COPD and chronic bronchitis

We used several items from the ATS questionnaire to evaluate chronic bronchitis, including the ATS diagnosis criteria as chronic phlegm most of the days at least three months a year during two consecutive years [16]. Although more recent definitions based on Global Initiative for Chronic Obstructive Lung Disease (GOLD) [17] defined COPD as a FEV_1_/FVC<70% and not necessarily FEV_1_<80% predicted, FEV_1 _values refer to post-bronchodilator FEV_1_. In this study, COPD was defined as both FEV_1_<80% predicted (referring to pre-bronchodilator values) and FEV_1_/FVC<70% to avoid possible reversible FEV1 value after bronchodilator test.

### Occupational exposure assessment

Occupational exposures were assessed in two different ways. First, subjects were asked about their current job (previous jobs were not asked for in the questionnaire). Subjects were classified according to their current occupation into broader occupational groups. Textile, metal and construction industries were considered as separate groups, while other blue-collar (potentially exposed) jobs were joined into one group due to the limited numbers of subjects for each job. Potentially high-risk jobs were compared with a large reference group of presumably non-exposed white-collar jobs (including civil servants, administrative, and clerical workers). Subjects that had retired or were unemployed were not included in these analyses.

Second, lifetime occupational exposures to dusts, gases and fumes was assessed directly by self-report using items from the standardised ATS questionnaire [14]. We defined lifetime occupational exposure to dust as an affirmative answer to the question: "Have you ever worked for a year or more in any dusty job?"; and lifetime occupational exposure to fumes/gases as an affirmative answer to question: "Have you ever been exposed (for a year or more) to gas or chemical fumes in your work?" Subjects that answered affirmatively to at least one of these two questions were subsequently asked in which job(s) this it had occurred, and what the duration of this exposure was.

### Statistical analysis

Analyses were conducted using the statistical package Stata version 6.0 (Stata Corporation, College Station, Texas, USA). Associations between respiratory symptoms, airflow obstruction, current occupation and lifetime occupational exposure were evaluated by calculating odds ratios (ORs) with 95% Confidence Interval (CI), using logistic regression analysis adjusting for sex, age and smoking status. Associations between reported occupational exposures and lung function variables were evaluated using linear regression analysis adjusting for sex, age, standing height and smoking status.

## Results

The majority of men in this population were smokers, whereas the majority of women had never smoked (table 1). Symptoms consistent with chronic bronchitis and obstructive lung disease were common, and more prevalent in men as compared to women. The prevalence of COPD was 10% in men and 4% in women.

**Table 1:** Demographic and respiratory health characteristics of the study population (N = 576)

	Men‡	Women‡
Total number	280 (100%)	296 (100%)
Age 20 to 44 years	135 (48.2%)	145 (49.0%)
Age 45 to 70 years	145 (51.8%)	151 (51.0%)
Current smokers	145 (51.8%)	62 (20.9%)
Ex-smokers	76 (27.1%)	31 (10.5%)
Never smokers	59 (21.1%)	203 (68.6%)
Chronic cough*	69 (24.6%)	24 (8.1%)
Chronic phlegm*	59 (21.1%)	10 (3.4%)
Wheezing during the last year	127 (45.4%)	93 (31.4%)
Wheezing apart from cold	92 (32.9%)	38 (12.8%)
Persistent wheezing†	32 (11.4%)	18 (6.1%)
Standing height (m)▫	1.70 (0.08)	1.57 (0.07)
FEV_1 _(L)▫	3.50 (0.88)	2.65 (0.59)
FEV_1 _to FVC ratio (%)▫	75.5 (9.2)	79.1 (6.5)
MMEF (L/s)▫	3.29 (1.46)	2.81 (0.95)
FEV_1 _<80% of predicted▫	42 (16.7%)	21 (8.5%)
FEV_1 _to FVC ratio <70%▫	49 (19.5%)	24 (9.8%)
FEV_1_<80% of predicted and FEV_1_/FVC<70%▫	26 (10.4%)	10 (4.1%)

‡Number (%) or mean (standard deviation) are given

▫Number of subjects with lung function data: 497 (251 men and 246 women)

* Most of the days at least three months a year during two consecutive years

† Wheezing most of the days or night

Four hundred and eight (71%) individuals were occupationally active at the time of the survey (table 2). Most of them (43%) were working in white-collar jobs, including civil servants, administrative and clerical workers. The distribution of occupation differed by sex. Textile industry was the most common blue-collar occupation in women (8.7%) and the second in men (10.2%) after the metal industry (11.5%). The category of remainder blue-collar jobs comprised a large variety of occupations for both sexes.

The prevalence of respiratory symptoms tended to be higher in blue-collar workers than in white-collar workers, although most associations did not reach statistical significance (table 2; see additional file 1). A reduced FEV_1 _was associated with work in the textile industry, and possibly also with construction work. Having evidence for airways obstruction (i.e., a low FEV_1 _to FVC ratio) was not apparently associated with current occupation. The highest risk for COPD was seen among workers in the construction industry.

Lifetime occupational exposure to dust, fumes or gases was reported by 52% of the subjects, being predominated by dust exposure (table 3). Reported exposure to dust was somewhat higher in men. Textile industry was the predominant occupation related to reported exposures in women, and the second most frequent job in exposed men after the metal industry.

**Table 3:** Reported lifetime occupational exposures and related industries (N = 576)

	Men	Women
Total number (%)	280 (100%)	296 (100%)
Lifetime occupational exposure to		
Dust	144 (51%)	110 (37%)
Fumes or gases	84 (30%)	25 (8%)
Dust, fumes or gases, or both	176 (63%)	122 (41%)
Reported jobs in relation to exposures		
Textile industry	41 (23%)	76 (62%)
Metal industry	49 (28%)	8 (7%)
Construction	28 (16%)	1 (1%)
Chemical industry	9 (5%)	13 (11%)
Other	49 (28%)	24 (20%)

The prevalence rates of chronic respiratory symptoms were higher in subjects reporting occupational exposures (table 4; see additional file 2). These associations were more pronounced in never-smokers, while wheezing was also associated with occupational exposures in ex-smokers. Airflow obstruction was not significantly related to occupational exposures, although elevated risks were found among never-smokers and ex-smokers. When analyses were adjusted for pack-years smoked instead of smoking status, results did not change (data not shown). Findings for men and women were comparable. After excluding asthmatics from analyses, Odds Ratios did not change significantly (results not shown).

Linear regression analyses showed overall non-significantly lower respiratory function indices in individuals exposed to dust, fumes or gases (table 5). The effects on lung function were more pronounced for individuals exposed at least 15 years, than for individuals exposed less than 15 years. For all presented analyses, risk estimates were similar when using exposure to dust or exposure to fumes/gases (results not shown).

**Table 5:** Associations between reported lifetime occupational exposure to dust, gases or fumes and lung function (N = 497)

	Ever exposed	Exposure duration <15 years	Exposure duration ≥ 15 years
Exposure to dust, fumes or gases (n)	268 (54%)	136 (27%)	132 (27%)
FEV_1 _(mL)‡	-33 (-117 to 51)	+3 (-95 to 100)	-80 (-186 to 26)
FEV_1 _to FVC ratio (%)‡	-1.1 (-2.3 to 0.1)	-0.6 (-2.1 to 0.8)	-1.7 (-3.3 to -0.2)
MMEF (mL/s)‡	-57 (-237 to 124)	+13 (-192 to 218)	-163 (-397 to 71)

‡ Linear regression coefficients (95% confidence intervals) denote mean differences in lung function parameters between exposed and never exposed individuals (n = 229); adjusted for sex, age, height and smoking status

## Discussion

In this study evaluating a general population sample with a high prevalence of lifetime occupational exposure to dust, fumes and gases (52%), we found an association of these exposures with chronic bronchitis and airflow obstruction independently of smoking status. Lung function impairment was related with duration of exposure. An increased risk for chronic bronchitis and airflow obstruction was found among workers in the textile industry that was the most prevalent current occupation in this population.

Several studies have shown an association between chronic bronchitis and occupational exposure in community-based studies [18-21], but the association with airflow obstruction independently of smoking is less clear [8]. One population-based study found that the effect of mineral dust exposure on chronic bronchitis symptoms was more apparent among current smokers [22]. We found an association between occupational exposures after adjusting by smoking status or pack-years of smoking, but residual confounding by smoking could have biased these results. Results, however, persisted when we limited the analysis among never smokers ruling out this possibility.

We found a negative effect of prolonged occupational exposure on lung function similar to other studies in industrial populations [11,23,24]. The current cross-sectional community-based study involved a wide spectrum of the population, implicating a wide variety of jobs, and selection bias due to the healthy worker effect is probably lower than in industry-based studies [12]. On the other hand, assessment of exposure in our study is through self-report and it is possible that exposure has been over-reported among symptomatic subjects. However, this study of respiratory symptoms and airflow obstruction was not aimed specifically to establish the occupational role in respiratory diseases, and the influence of over-reporting in the results is not likely to play a major role. Population specific job-exposure matrices (JEMs) may be useful in the assessment of occupational exposure but require a large number of subjects per job. Therefore, the use of self-reported exposures has been frequently suggested for general population studies [21].

The most important industry implicated in our exposed population is the textile industry. Although this industry is the most prevalent as current work (7%), it was more important twenty to thirty years ago in this population area with 20% of the population reporting textile industry as lifetime exposure. The observed association between lifetime exposure and lung function impairment reflects to a large extent this historical exposure, above all among women. An association between employment in the textile industry and a steeper FEV_1 _decline has been described previously [25]. It has also been suggested that the occurrence of respiratory symptoms represents the earliest response and a risk factor for subsequent loss of pulmonary function [26]. The majority of our study population were younger than 50 years, and exposure duration was probably not long enough for airflow limitation to become apparent.

We evaluated the effect of occupational exposures among never smokers to avoid a potential residual confounding effect of smoking. Risks for respiratory symptoms and airflow limitation among never smokers were higher than risks among current smokers. This pattern was unexpected on the basis of findings from other community-based studies [8], and a postulated, but not clearly demonstrated interaction between smoking and occupational exposures in the relationship with COPD. Differential misclassification of occupational exposure or symptom reporting among non-smokers compared to smokers is *a priori *not impossible, but has not been reported often. Specific jobs related to wood and textile industry have found a similar pattern for occupational exposures and smoking [27,28], which could involve immunological [9] and genetic factors [29]. A final explanation for our findings could be that the effect of occupational exposures, which is proportionally smaller to that of smoking, is easier to detect among never-smokers.

There are a number of limitations in our study that should be considered. First, we did not collect full (lifetime) occupational history. This could have resulted in differential misclassification of industry if individuals with obstructive lung disorders had changed job and moved to the white-collar category. This could have biased the risk estimates associated with job classification towards the null, thus observed effects could have been underestimated. Nevertheless, experience with studies in this area with the same age distribution suggested that for the vast majority of subjects current job reflects longest held job. In addition, we were not able to identify subjects who never had worked and therefore these could not be excluded from the analyses using (reported) lifetime exposures. One could argue that those who had never worked had no "exposure opportunity", but the approach to group never workers with "white collar workers" or others without any history of relevant occupational exposures is conservative and allows interpretation at the population level.

Second, we used self-reports to assess lifetime occupational exposures to dusts and gases/fumes. A major concern about this approach is the potential for differential misclassification if symptomatic individuals are more likely to recall and report occupational airborne exposures, which may result in a bias away from the null [30]. The interpretation of self-reported exposures in international studies may be problematic since the validity may be different for different countries [30]. Nevertheless, comparisons made within defined geographical areas show reasonable agreement with JEMs, and large differences in validity between symptomatics and non-symptomatics have not been found [30,31]. Self-reports allow for variation in exposures within job titles, providing an advantage over the use of JEMs.

## Conclusion

Occupational lifetime exposure to dust, fumes and gases evaluated in the general population was associated with chronic bronchitis and airflow obstruction. The most frequent job associated with these exposures was the textile industry. The effect of occupational exposure on lung function was related with longer duration of exposure, independently of smoking status. Longitudinal community-based studies with adequate assessment of occupational exposure will be necessary to assess the role of occupational exposures in development of COPD, independently of smoking exposure.

## Abbreviations

COPD: Chronic Obstructive Pulmonary Disease

FEV_1_: Forced Expiratory Volume at one second

ATS: American Thoracic Society

OR: Odds Ratio

CI: Confidence Interval

FVC: Forced Vital Capacity

JEM: Job Exposure Matrix

## Competing interests

The author(s) declare that they have no competing interests.

## Authors' contributions

AJ participated in the design of study, carried out the interviewers and lung function tests, performed the statistical analysis and interpretation of data, and wrote the manuscript. JPZ participated in the design and classification of occupational exposures, participated in analysis and interpretation of the data and was involved in drafting and revising the manuscript for important intellectual content. MK participated in design and interpretation of the data and was involved in drafting and revising the manuscript for important intellectual content. AF and AM participated in design and were involved in drafting and revising the manuscript for important intellectual content.

## Supplementary Material

Additional file 1 Contains Table 2 (landscape format). Click here for file

Additional file 2 Contains Table 4 (landscape format). Click here for file

## References

[B1] National Heart Lung and Blood Institute and World Health Organization (2001). Global initiative for chronic obstructive lung disease: a collaborative project of the National, Hearth, Lung, and Blood Institute and the World Health Organization.

[B2] Lee PN, Fry JS, Forey BA (1990). Trends in lung cancer, chronic obstructive lung disease, and emphysema death rates for England and Wales 1941–85 and their relation to trends in cigarette smoking. Thorax.

[B3] Venables KM, Chan-Yeung M (1997). Occupational asthma. Lancet.

[B4] Pride NB, Burrows B, Carverley P, Pride N (1995). Development of impaired lung function: natural history and risk factors. Chronic obstructive pulmonary disease.

[B5] Trupin L, Earnest G, San Pedro M, Balmes JR, Eisner MD, Yelin E, Katz PP, Blanc PD (2003). The occupational burden of chronic obstructive pulmonary disease. Eur Respir J.

[B6] Balmes J, Becklake M, Blanc P, Henneberger P, Kreiss K, Mapp C, Milton D, Schwartz D, Toren K, Viegi G (2003). American Thoracic Society Statement: occupational contribution to the burden of airway disease. Am J Respir Crit Care Med.

[B7] Oxman AD, Muir DCF, Shannon HS, Stock SR, Hnizdo E, Lange HJ (1993). Occupational dust exposure and chronic obstructive pulmonary disease. A systematic overview of the evidence. Am Rev Respir Dis.

[B8] Zock JP, Sunyer J, Kogevinas M, Kromhout H, Burney P, Antó JM (2001). Occupation, chronic bronchitis, and lung function in young adults. An international study. Am J Repir Crit Care Med.

[B9] Hendrick DJ (1996). Occupation and chronic obstructive pulmonary disease (COPD). Thorax.

[B10] Ulvestad B, Bakke B, Eduard W, Kongerud J, Lund MB (2001). Cumulative exposure to dust causes accelerated decline in lung function in tunnel workers. Occup Environ Med.

[B11] Post W, Heederik D, Houba R (2000). Decline in lung function related to exposure and selection processes among workers in the grain processing and animal feed industry. Occup Environ Med.

[B12] Burge PS (1994). Occupation and chronic obstructive pulmonary disease (COPD). Eur Respir J.

[B13] Jaen A, Ferrer A, Ormaza I, Rue M, Domingo C, Marin A (1999). Prevalencia de bronquitis crónica, asma, y obstrucción al flujo aéreo en una zona urbano-industrial de Cataluña. (In Spanish, with English summary). Arch Bronconeumol.

[B14] Ferris BG (1978). Epidemiology standardization project. II. Recommended respiratory disease questionnaires for use with adults and children in epidemiologic research. Am Rev Respir Dis.

[B15] Roca J, Sanchis J, Agustí-Vidal A, Segarra F, Navajas D, Rodriguez-Roisin R, Casan P, Sans S (1986). Spirometric reference values from a Mediterranean population. Bull Eur Physiopathol Respir.

[B16] American Thoracic Society (1987). Standards for the diagnosis and care of patients with chronic obstructive pulmonary disease (COPD) and asthma. Am Rev Respir Dis.

[B17] Pauwels RA, Buist AS, Calverley PM, Jenkins CR, Hurd SS, GOLD Scientific Committee (2001). Global strategy for the diagnosis, management, and prevention of chronic obstructive pulomary disease. NHLBI/WHO Global obstructive lung disease (GOLD) workshop summary. Am J Respir Crit Care Med.

[B18] Kryzanowski M, Kauffmann F (1988). The relation of respiratory symptoms and ventilatory function to moderate occupational exposure in a general population. Int J Epidemiol.

[B19] Heederik D, Kromhout H, Burema J, Biersteker K, Kromhout D (1990). Occupational exposure and 25-year incidence rate of non-specific lung disease: The Zutphen Study. Int J Epidemiol.

[B20] Sunyer J, Kogevinas M, Kromhout H, Anto JM, Roca J, Tobias A, Vermeulen R, Payo F, Maldonado JA, Martinez-Moratalla J, Muniozguren N (1998). Pulmonary ventilatory defects and occupational exposures in a population-based study in Spain. Am J Respir Crit Care Med.

[B21] Post WK, Heederik D, Kromhout H, Kromhout D (1994). Occupational exposures estimated by a population specific job exposure matrix and 25 year incidence rate of chronic non-specific lung disease (CNSLD): the Zutphen Study. Eur Respir J.

[B22] De Meer G, Kerkhof M, Kromhout H, Schouten JP, Heederik D (2004). Interaction of atopy and smoking on respiratory effects of occupational dust exposure: a general population-based study. Environ Health.

[B23] Sobaszek A, Boulenguez C, Frimat P, Robin H, Haguenoer JM, Edme JL (2000). Acute respiratory effects of exposure to stainless steel and mild steel welding fumes. J Occup Environ Med.

[B24] Bradshaw LM, Fishwick D, Slater T, Pearce N (1998). Chronic bronchitis, work related respiratory symptoms, and pulmonary function in welders in New Zealand. Occup Environ Med.

[B25] Christiani DC, Ye TT, Zhang S, Wegman DH, Eisen EA, Ryan LA, Olenchock SA, Pothier L, Dai HL (1999). Cotton dust and endotoxin exposure and long-term decline in lung function: results of a longitudinal study. Am J Ind Med.

[B26] Wang XR, Pan LD, Zhang HX, Sun BX, Dai Hl, Christiani DC (2002). Follow-up study of respiratory health of newly-hired female cotton textile workers. Am J Ind Med.

[B27] Schlunssen V, Schaumburg I, Ebbe T, Mikkelsen AB, Sigsgaard T (2002). Respiratory symptoms and lung function among Danish woodworkers. J Occup Ennviron Med.

[B28] Niven RM, Fletcher AM, Pkickering CAC, Fishwick D, Warburton CJ, Simpson JCG, Francis H, Oldham LA (1997). Chronic bronchitis in textile workers. Thorax.

[B29] Suadicani P, Hein HO, Meyer HW, Gyntelberg F (2001). Exposure to cold and draught, alcohol consumption, and the NS-phenotype are associated with chronic bronchitis: an epidemiological investigation of 3387 men aged 53–75 years: the Copenhagen Male Study. Occup Environ Med.

[B30] De Vocht F, Zock JP, Kromhout H, Sunyer J, Antó JM, Burney P, Kogevinas M (2005). Comparison of self-reported occupational exposure with a job exposure matrix in an international community-based study on asthma. Am J Ind Med.

[B31] Blanc PD, Eisner MD, Balmes JR, Trupin L, Yelin EH, Katz PP (2005). Exposure to vapors, gas, dust, or fumes: assessment by a single survey item compared to a detailed exposure battery and a job exposure matrix. Am J Ind Med.

