# Supplementary material for: Occupation, smoking, and chronic obstructive respiratory disorders: a cross sectional study in an industrial area of Catalonia, Spain
Source: Environ Health. 2006 Feb 14;5:2. doi: 10.1186/1476-069X-5-2 (PMC1388209; doi:10.1186/1476-069X-5-2)
Supplement: Additional file 1 — Contains Table 2 (landscape format). [file 1476-069X-5-2-S1.doc]

Table 2: Associations between current occupation, respiratory symptoms and airflow obstruction (N=408*)

|  | White-collar | Textile | Metal‡ | Construction‡ | Remainder  blue-collar |
| --- | --- | --- | --- | --- | --- |
| Total number | 175 | 39 | 28 | 20 | 146 |
| Women: n (%) | 81 (46%) | 15 (38%) | 1 (4%) | 0 (−) | 76 (52%) |
| Current smokers: n (%) | 74 (42%) | 20 (51%) | 15 (54%) | 11 (55%) | 58 (40%) |
| Ex-smokers: n (%) | 33 (19%) | 7 (18%) | 8 (29%) | 8 (40%) | 20 (14%) |
| Chronic cough†+ | 1.0 (Referent) | 1.7 (0.7 to 4.4) | 2.0 (0.7 to 6.1) | 2.0 (0.6 to 6.7) | 1.2 (0.6 to 2.4) |
| Chronic phlegm†+ | 1.0 (Referent) | 2.3 (0.8 to 6.7) | 1.1 (0.3 to 4.0) | 2.5 (0.7 to 8.9) | 2.7 (1.2 to 6.0) |
| Wheezing during the last year+ | 1.0 (Referent) | 1.1 (0.5 to 2.4) | 1.9 (0.7 to 5.0) | 2.5 (0.8 to 7.6) | 1.4 (0.8 to 2.2) |
| Wheezing apart from cold+ | 1.0 (Referent) | 1.5 (0.6 to 3.4) | 2.2 (0.8 to 5.9) | 3.7 (1.3 to 11) | 1.4 (0.8 to 2.5) |
| FEV_1_ <80% of predicted▫+ | 1.0 (Referent) | 3.3 (1.1 to 10) | 1.3 (0.3 to 4.9) | 3.2 (0.7 to 14) | 1.8 (0.8 to 4.4) |
| FEV_1_ to FVC ratio <70%▫+ | 1.0 (Referent) | 1.5 (0.5 to 5.0) | 0.5 (0.1 to 2.0) | 1.8 (0.4 to 7.6) | 0.9 (0.4 to 2.2) |
| FEV_1_<80% pred. and FEV_1_/FVC<70%▫+ | 1.0 (Referent) | 1.4 (0.3 to 6.1) | 0.8 (0.2 to 3.8) | 3.9 (0.9 to 18) | 1.1 (0.3 to 3.4) |

* 168 subjects (45 men and 123 women) were either housewife, unemployed or retired, and were not included in this analysis

▫ Number of subjects with lung function data: 363 (159+34+27+16+127)

+ Odds Ratios (95% confidence intervals) relative to white collar workers, adjusted for sex, age and smoking status

† Most of the days at least three months a year during two consecutive years

‡ Analyses include exclusively men (one lifetime non-smoking woman working in the metal industry was excluded)
