# Supplementary material for: Occupation, smoking, and chronic obstructive respiratory disorders: a cross sectional study in an industrial area of Catalonia, Spain
Source: Environ Health. 2006 Feb 14;5:2. doi: 10.1186/1476-069X-5-2 (PMC1388209; doi:10.1186/1476-069X-5-2)
Supplement: Additional file 2 — Contains Table 4 (landscape format). [file 1476-069X-5-2-S2.doc]

**Table 4: Associations between reported lifetime occupational exposure to dust, gases or fumes, respiratory symptoms
and airflow obstruction**

Odds Ratios (95% confidence intervals) relative to never exposed (n=278); adjusted for for sex, age, and smoking status where applicable

|  | All | Never smokers | Current smokers | Ex-smokers |
| --- | --- | --- | --- | --- |
| Total number | 576 | 262 | 207 | 107 |
| Exposure to dust, fumes or gases | 298 (52%) | 111 (42%) | 129 (61%) | 58 (57%) |
| Chronic cough* | 1.8 (1.1 to 3.0) | 3.4 (1.1 to 10) | 1.2 (0.6 to 2.5) | 2.1 (0.7 to 6.9) |
| Chronic phlegm* | 2.0 (1.1 to 3.7) | 3.7 (0.9 to 15) | 1.6 (0.7 to 3.6) | 1.9 (0.5 to 7.2) |
| Wheezing during the last year | 1.7 (1.1 to 2.4) | 2.1 (1.2 to 3.7) | 0.9 (0.5 to 1.7) | 5.0 (1.5 to 17) |
| Wheezing apart from cold | 2.0 (1.3 to 3.1) | 2.9 (1.3 to 6.3) | 1.1 (0.6 to 2.2) | 4.5 (1.5 to 14) |
| Persistent wheezing† | 2.0 (1.0 to 3.9) | 4.3 (1.1 to 17)‡ | 1.3 (0.5 to 3.3) | 1.8 (0.4 to 7.5) |
| FEV_1_ <80% of predicted▪ | 1.5 (0.8 to 2.8) | 1.9 (0.7 to 4.6) | 0.7 (0.3 to 1.9) | 6.5 (0.8 to 55) |
| FEV_1_ to FVC ratio <70%▪ | 1.2 (0.7 to 2.2) | 2.2 (0.9 to 5.4) | 0.6 (0.2 to 1.6) | 0.8 (0.2 to 3.1) |
| FEV_1_<80% pred. and FEV_1_/FVC<70%▪ | 1.2 (0.7 to 2.2) | 1.6 (0.4 to 6.3) | 0.5 (0.1 to 1.6) | 4.8 (0.5 to 42) |

▪ Number of subjects with lung function data: 497 (210+193+94)

* Most of the days at least three months a year during two consecutive years

† Wheezing most of the days or nights

‡ Analyses included exclusively women (n=203)
